# The effect of cannabinoid receptor agonist WIN 55,212–2 on anxiety‐like behavior and locomotion in a genetic model of absence seizures in the elevated plus‐maze

**DOI:** 10.1111/cns.13848

**Published:** 2022-04-26

**Authors:** Daniel Cassar, Manuela Radic, Maurizio Casarrubea, Vincenzo Crunelli, Giuseppe Di Giovanni

**Affiliations:** ^1^ Laboratory of Neurophysiology Department of Physiology and Biochemistry Faculty of Medicine and Surgery University of Malta Msida Malta; ^2^ Laboratory of Behavioral Physiology Human Physiology Section “Giuseppe Pagano” Department of Biomedicine, Neuroscience and Advanced Diagnosistics (BIND) University of Palermo Palermo Italy; ^3^ Neuroscience Division School of Biosciences Cardiff University Cardiff UK

## Abstract

GAERS and NEC rats were treated with cannabinoid 1/2 receptor agonist WIN 55,212‐2 2 mg/kg and tested on the Elevated Plus‐Maze.

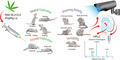

## 1 INTRODUCTION

Childhood Absence Epilepsy (CAE) is a form of genetically determined generalized epilepsy, with complex multifactorial inheritance and onset in children between 4 and 12 years of age. It accounts for 10%–15% of all childhood epilepsies. It is characterized by nonconvulsive absence seizures (ASs) that are sudden, relatively brief gaps of consciousness concurrent with a lack of voluntary movements and 2.5–4 Hz spike and wave discharges (SWDs) generalized in the electroencephalogram (EEG).^1^ Moreover, ASs are the only clinical symptom in CAE, although patients can present them simultaneously with other seizure types in different epilepsies. ASs in children and teenagers were in the past considered relatively benign because of their nonconvulsive nature and high remittance rate in early adulthood. However, recent studies in large cohorts of drug naïve CAE patients have now conclusively demonstrated that 30% of children with ASs are pharmacoresistant, while 60% suffer from various neuropsychiatric disorders.^1^ These comorbidities include anxiety, depression, as well as attention and learning deficits that may precede epilepsy diagnosis, persist, and even be aggravated after full pharmacological control of the seizures^1^). A recent meta‐analysis has shown a strong bidirectional relationship between ASs and anxiety both in humans and in animal models.^2^ The Genetic Absence Epilepsy Rats from Strasburg (GAERS), presenting both ASs and neuropsychiatric comorbidities,^1^, ^2^ are a useful tool to further our understanding of the link between anxiety and epilepsy in CAE.

**FIGURE 1:**
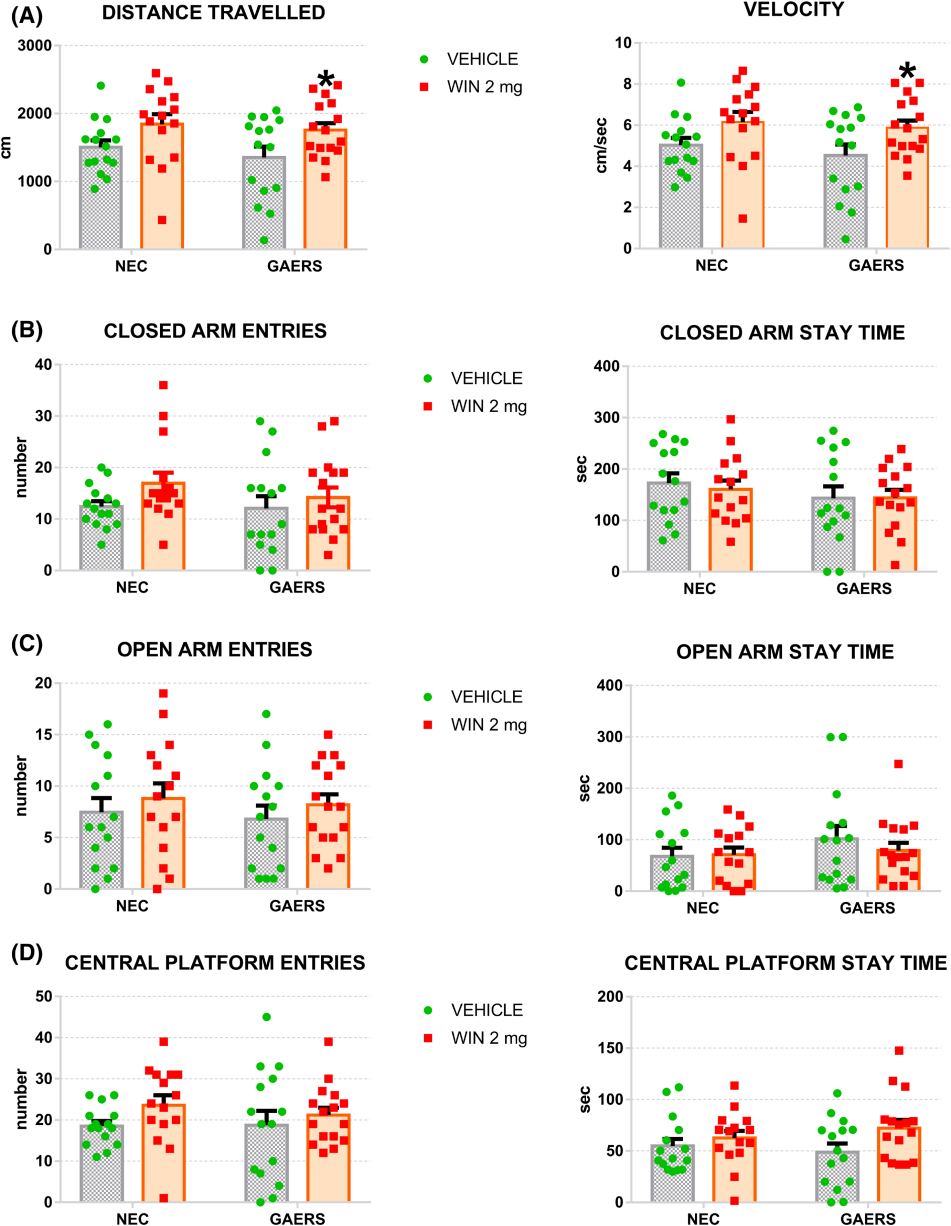
Effect of the cannabinoid CB1/2 receptor agonist WIN 55,212‐2 on GAERS and NEC rats on Elevated Plus‐Maze performance. Sixty NEC and GAERS rats were randomly assigned to 4 groups: NEC treated with vehicle (*n* = 15), NEC treated with 2 mg/kg i.p., WIN 55,212‐2 (WIN; *n* = 15), GAERS treated with vehicle (*n* = 15), GAERS treated with 2 mg/kg i.p., WIN 55,212‐2 (*n* = 15). After 30 min of WIN/vehicle administration, GAERS and NEC were placed in the center of the elevated plus‐maze (EPM), facing the open arm opposite to the experimenter. The Distance Travelled, the Velocity (A), the number of Closed Arm Entries, the Closed Arm Stay Time (B), the Open Arm Entries, the Open Arm Stay Time (C) and the Central Platform Entries and Central Platform Stay Time (D) were computed automatically by commercially available software. Two‐way ANOVA followed by Fisher's PLSD post hoc test for multiple comparisons. *p < 0.05 between GAERS and NEC group

We focused our work on the endocannabinoid system (ECS) since recent preclinical evidence indicates that its activation may have an antiabsence action representing a new target for CAE treatment.^3^, ^4^, ^5^ Morover, ECS has been involved in the etiopathogenesis of anxiety although opposite influences of the cannabinoids have been reported.^6^ It is likely that ECS has a unique regulatory capacity for maintaining emotional homeostasis and the effect of its activation depend on the baseline conditions.^6^ Therefore, to shed some light on CAE comorbidities and their modulation of by the ECS, we investigated the effect of the acute systemic administration of WIN 55,212‐2, a synthetic highly potent, full‐acting cannabinoid 1/2 receptor (CB1/2R) agonist on anxiety‐like behavior in GAERS and their non‐epileptic control (NEC) rats. Male GAERS and NEC rats (3–5 months old) were obtained from a colony bred at the University of Malta. All GAERS but not NEC rats showed spike and wave discharges (SWDs) in the EEG recordings as observed in other colonies^7^ (data not shown). Animals were housed in a 12:12 light cycle (lights on at 07.00 a.m. and off at 07.00 p.m.). All animal procedures were approved and carried out following University of Malta ethical guidelines and in conformity with Maltese and international laws and policies (EU Directive, 2010/63/EU for animal experiments). A moderate‐high dose of WIN 55,212–2 of 2 mg/kg was intraperitoneally (i.p.) administered to examine the behavioral thigmotaxis and explorative response in the Elevated Plus‐Maze (EPM) apparatus, commonly used to evaluate anxiety response to a novel environment in rodents. Animals before the test were handled and habituated to the experimental room and tested under dim lighting (about 50 lux) during the dark phase. WIN 55,212‐2 was freshly dissolved in a vehicle solution (2 ml/kg) made of 5% PEG‐400, 5% Tween 80 in saline. After 30 min of drug/vehicle administration, GAERS and NEC were submitted to the EPM. The rats were positioned on the central platform with their tail pointed toward the closed arm nearest to the experimenter. Rats were left free to explore the EPM for 5 min while a video camera placed orthogonally to the maze recorded from above (see Graphical Abstract). The rat behavior was video‐recorded. The Distance Travelled, the Velocity, the number of Closed Arm Entries, the Closed Arm Stay Time, the Open Arm Entries, the Open Arm Stay Time were computed by an automated system (Noldus Ethovision 10.0; Noldus Information Technologies). Two‐way ANOVA (Graphpad Prism 5 03, GraphPad Software Inc.) revealed significant main effect of pharmacological treatment (strain x treatment) only for Distance (F (1, 57) = 8.156 *p* < 0.001) and Velocity (F (1, 57) = 8.037; *p* < 0.001). Unexpectedly, vehicle‐treated GAERS (*n* = 15) resulted in a similar anxious state to NEC control rats (*n* = 15) (Figure 1). Moreover, the two strains (*n* = 15, each) continued to show similar anxiety‐like behavior after the pharmacological activation of CB1/2R with 2 mg/kg WIN 55,212‐2 treatment (Figure 1). As far the locomotor activity is concerned, GAERS and NEC treated with vehicle did not differ for travel distance or velocity while WIN 55,212‐2 increased traveled distance (*p* < 0.05) and the velocity of ambulation (*p* < 0.05) in GAERS compared to NEC (Figure 1A).

Different colonies of GAERS and NEC have been established in different countries, and different results have been obtained depending on the colony examined. For instance, our findings on control GAERS and NEC agree with the original evidence by Marescaux's group^8^ or more recent EPM findings from Saskatchewan^9^ showing that male GAERS are equally anxious as NEC. On the other hand, our results contrast with EPM results obtained from the Strasburg colony^10^ or from the evidence from Melbourne showing GAERS rats spending less time in open arms and making more open arm entries. In addition, we also could not reveal an effect of strain on the distance traveled in the EPM that instead it was previously reported to be decreased in GAERS compared with NEC.^9^ Different breeding and animal housing conditions and early‐life stress determine the anxious state of adult animals and epilepsy severity and expression. Therefore, the lack of strain difference in locomotion and anxiety‐like behavior observed in our colony may depend on the specific environmental conditions in Malta. The CB1/2R agonist WIN 55,212‐2 administration did not modify the anxiety‐like response in GAERS and NEC submitted to the EPM task but induced hyperlocomotion. The latter is quite surprising, as high doses of WIN‐55,212‐2 caused instead hypolocomotion on non‐epileptic rats exposed to EPM.^11^ This hyperactivity induced by WIN 55,212‐2 in GAERS might depend on the decrease in accumbal dopamine transporter and D2R levels^12^ and/or the ECS impairment^3^ observed in this strain. Nevertheless, as we only recorded the motor behavior for the 5 min of the EPM test, we cannot exclude a decline in locomotor activity over a longer interval of time.

In conclusion, in our conditions, GAERS and NEC vehicle‐treated rats showed unexpectedly a similar level of anxiety. Nevertheless, differentially from previous evidence, the animals from this study were restrained in order to receive the i.p. vehicle administration and this might have had an emotional impact. Interestingly, for the first time, a strain‐dependent responsiveness to cannabinoid treatment consisting in hypermotility was observed in GAERS compared with NEC. Surprisingly, activation of the CB1Rs did not modify the anxiety‐like behavior in either of the two strains. The present findings extend previous evidence indicating an ECS alteration in animal models of absence seizures,^5^ although they do not suggest a possible therapeutic use of cannabinoids on comorbid anxiety in AE. Nevertheless, we cannot draw final conclusions from our results that need to be taken with caution and warrant further investigation.

## CONFLICT OF INTEREST

The authors declare that the research work was conducted in the absence of any commercial or financial relationships that could be construed as a potential conflict of interest.

## AUTHOR CONTRIBUTIONS

G.D.G conceived the study and designed the methodology and performed data interpretation. D.C. conducted all the laboratory‐based research and performed data interpretation. G.D.G., V.C., and M.C. wrote the manuscript and M.R., and D.C. prepared figures. G.D.G., M.C., and V.C. have reviewed and edited the manuscript. All authors have agreed to this manuscript submission for publication.

## Data Availability

The datasets generated during the current study are available from the corresponding authors on reasonable request.
